# Deciduous Teeth

**Published:** 1881-09

**Authors:** H. Cowie


### DECIDUOUS TEETH.

BY DR. H. COWIE.

*Read before the Michigan State Dental Association.*

Probably no portion of the Dental economy is less appreciated and understood by the public and even indeed by the members of our profession than the deciduous teeth, their care, importance, and particularly the treatment they require, and ought to have at our hands.

These little pearls, unless prematurely or improperly interfered with, are usually erupted in a regular arch, giving beauty and expression to the features, and making a perfect frame work for the proper proportion of the little jaws and integuments during early childhood. When we consider the value of their relation to the dental system in preserving the arch from contraction, and thereby preventing the irregular condition so often to be found of the permanent teeth; and how much the general health of the child depends on their retention until replaced by their successors—thus affording a proper and sufficient apparatus for the comminution of food, so that it may be assimilated and digested by the child, we can not overestimate their value, and cannot deprecate too highly the too frequent loss of these teeth by decay, and the too early loss by extraction to which they are subjected.

Is it not time that we turn our attention to them more than we have yet done and to try, if possible, to do better by them?

We must surely realize the importance of doing something more than we have yet succeeded in doing, when we see, as we all must do every day, the generally bad condition of the mouths presented to our notice, induced by the decay and loss of those temporary teeth brought about by the neglect and ignorance or want of care of both parent and dentist.

Parents, even those who give attention to their own teeth and to those of their children they may deem sufficiently advanced in years, even they are generally in too great a hurry to get rid of the baby teeth, and look upon them as mere obstructions to the second set. They come to us with their little ones only when compelled by the failure on their part, to stop the pain of toothache, by all the means in their power, and with the request that we extract the offender, and will hardly listen with any patience to our advice, that the tooth or teeth be kept, and something also done for the rest of the teeth that may require attention to prevent a like result from them in time, offering as a reason that they are only temporary and will be replaced by other and better teeth so soon. Then, too, how often we have to contend that the first permanent molars are not temporary teeth but belong to the second set. When we so tell them they will exclaim, "Oh, I know they are only first teeth, for I have watched the child's teeth and know they have not shed them." Of course it is our duty not to let them go until we have used reasonable efforts to instruct and convince them of the truth of what we have said, both in regard to the value of the temporary teeth, and particularly, the value the first permanent molars have to the future well being of the child, and to impress upon them the importance of having them examined by their dentist at regular periods.

We, as a profession, have much to learn in order to arrive at a proper understanding of these deciduous teeth, so that we may be able to properly treat and care for these neglected little teeth.

It is hard in any case to operate for children. They are so easily tired, and still more easily hurt; and with our present knowledge, unless we have a chance of attending to them before they require much done, it is almost a hopeless task when called to operate on these little ones when suffering from toothache. We are perplexed to know just what we ought to do, in order to give relief, and at the same time do what we ought to do for the future interests of the little ones under our care. For if we take out the teeth before the proper time we allow the jaws to contract, deprive them of what they need, and ought to use in the preparation of their food. If we destroy the nerve we are apt to cause more pain in the doing of it, and perhaps cause an attack of inflammation.

My object in this paper is, to, if possible, get some ideas on this much neglected topic, and would like very much to have it occupy a portion of your time, so that it may be discussed by the members of this society in all its bearings, so that we may improve in our treatment.

1st. Would like to hear from those present what filling material may be best to use.

2d. What may be the best method of treatment for aching temporary teeth, without a resort to extraction to cure the pain and save the teeth.

3d. Would also ask when nature sometimes fails to remove the temporary teeth, are we to extract when there may not be any indication of the permanent successor? You have all seen cases when perhaps only one of the temporary teeth may be left out of the entire set.

4th. Does not nature sometimes preserve deciduous teeth to fill the places of permanent ones?

Among the cares to be bestowed on the deciduous teeth during the progress of the second dentition is that of extraction at the proper time. Experience will teach that too early an extraction will be worse than too long delay.

It is a beautiful operation to correct a difficult case of irregularity of the teeth, but it is in my opinion a much more desirable thing to prevent its occurrence by giving the proper attention at the right time to the temporary teeth.

One more question and I will close—is it good practice to extract the two temporary laterals to give room to the permanent centrals when the laterals are loose and the centrals have not room to develope properly into position? I single out this one case particularly because it seems to me to be the key note, if I may so say, to the proper developement of those that follow.

*Transactions Mich. Dental Society.*

